# Chemicals Used in Plastic Materials: An Estimate of the Attributable Disease Burden and Costs in the United States

**DOI:** 10.1210/jendso/bvad163

**Published:** 2024-01-11

**Authors:** Leonardo Trasande, Roopa Krithivasan, Kevin Park, Vladislav Obsekov, Michael Belliveau

**Affiliations:** Department of Pediatrics, NYU Grossman School of Medicine, NewYork, NY 10016, USA; Department of Population Health, NYU Grossman School of Medicine, NewYork, NY 10016, USA; NYU Wagner Graduate School of Public Service, NewYork, NY 10012, USA; Defend Our Health, Portland, ME 04101, USA; Department of Medicine, NYU Grossman School of Medicine, NewYork, NY 10016, USA; Children’s Hospital of Philadelphia, Philadelphia, PA 19104, USA; Defend Our Health, Portland, ME 04101, USA

**Keywords:** cost, disease, perfluoroalkyl substances, phthalates, bisphenols, plastics

## Abstract

**Context:**

Chemicals used in plastics have been described to contribute to disease and disability, but attributable fractions have not been quantified to assess specific contributions. Without this information, interventions proposed as part of the Global Plastics Treaty cannot be evaluated for potential benefits.

**Objective:**

To accurately inform the tradeoffs involved in the ongoing reliance on plastic production as a source of economic productivity in the United States, we calculated the attributable disease burden and cost due to chemicals used in plastic materials in 2018.

**Methods:**

We first analyzed the existing literature to identify plastic-related fractions (PRF) of disease and disability for specific polybrominated diphenylethers (PBDE), phthalates, bisphenols, and polyfluoroalkyl substances and perfluoroalkyl substances (PFAS). We then updated previously published disease burden and cost estimates for these chemicals in the United States to 2018. By uniting these data, we computed estimates of attributable disease burden and costs due to plastics in the United States.

**Results:**

We identified PRFs of 97.5% for bisphenol A (96.25-98.75% for sensitivity analysis), 98% (96%-99%) for di-2-ethylhexylphthalate, 100% (71%-100%) for butyl phthalates and benzyl phthalates, 98% (97%-99%) for PBDE-47, and 93% (16%-96%) for PFAS. In total, we estimate $249 billion (sensitivity analysis: $226 billion-$289 billion) in plastic-attributable disease burden in 2018. The majority of these costs arose as a result of PBDE exposure, though $66.7 billion ($64.7 billion-67.3 billion) was due to phthalate exposure and $22.4 billion was due to PFAS exposure (sensitivity analysis: $3.85-$60.1 billion).

**Conclusion:**

Plastics contribute substantially to disease and associated social costs in the United States, accounting for 1.22% of the gross domestic product. The costs of plastic pollution will continue to accumulate as long as exposures continue at current levels. Actions through the Global Plastics Treaty and other policy initiatives will reduce these costs in proportion to the actual reductions in chemical exposures achieved.

In March 2022, the United Nations Environment Assembly announced plans for a global plastics treaty. Early negotiations over the subsequent year focused on the effects of plastics on oceans and wildlife, with human health barely mentioned [1]. More recently, governments are increasingly incorporating the effects of chemicals used in plastic materials as considerations. These include bisphenols used in polycarbonate plastics, polybrominated diphenylethers (PBDEs) used as flame retardant additives, phthalates used in polyvinyl chloride (PVC) plastics and perfluoroalkyl substances (PFAS) used as additives to polyethylene plastics or used as monomers in fluoroplastic polymers [2].

Resisting the focus on chemicals are those countries with economies substantially driven by fossil fuels [3]. Their governments are concerned about the costs of policy options that reduce plastic production, because many plastics are derived from these materials [4]. The United States, for example, produced 18% of all plastic produced globally in 2019, with a market share of $97.5 billion [5].

The social costs of disease and disability in the United States due to PBDEs, phthalates, and bisphenols [6, 7], as well as PFAS [8], are very large, on the order of $400 billion annually. A more recent study by the Minderoo-Monaco Commission suggests even higher costs, more than $900 billion per year [9]. But the commission mistakenly assumes that PBDEs, phthalates, and bisphenols are used only in plastic materials, when these chemicals are also used in nonplastic applications, including solvents and ceramics [10]. It also propagated a mathematical error in the population affected by phthalate exposure, overestimating attributable mortality by a factor of two [7].

To accurately inform the tradeoffs involved in the ongoing reliance on plastic production as a source of economic productivity in the United States, we calculated the attributable disease burden and cost due to chemicals used in plastic materials in 2018.

## Materials and Methods

### Overall Approach

The present manuscript builds on previous analyses led by 2 of the authors (L.T. and V.O.) [6-8], which we updated and aligned to represent disease burden and costs due to PBDEs, phthalate, bisphenol, and PFAS exposure in 2018. As the methods have been published previously, we provide here a brief synopsis of the key methodological updates, and refer the reader to a detailed supplement [11] that describes the calculations in detail. As probabilities of causation were not available for all the diseases and dysfunctions considered, they were not propagated as performed for endocrine-disrupting chemicals in the United States.

Subsequent sections explain the methodology used to estimate plastics-related fractions (PRFs) of disease and disability due to PBDEs, phthalate, bisphenol, and PFAS exposures.

### Defining Plastic-related Fractions

We define a product as a plastic if it consists of synthetic and semisynthetic materials made of polymeric substances. We include all thermoplastics (both resin and fiber), thermosets (including epoxy resins, polyurethane [PUR] resins, and phenoplastics), and elastomers (including PUR, neoprene, and silicone). We include polymers if they are synthetic (polymerized from either petrochemical or biobased monomers), or semisynthetic (natural polymers such as cellulose and natural rubber that are chemically modified, such as rayon), but do not include naturally occurring polymers (eg, cellulose, 100% cotton, natural rubber).

For each chemical, we compiled sources that provide a list of all its uses and that include quantities or proportions of total chemical production allocated to each use. Sources include industry reports; reports produced by national and international governing bodies, including the European Chemicals Agency (ECHA); and peer-reviewed publications. Search terms used to identify relevant gray literature and peer-reviewed publications include combinations of the chemical's common name (eg, “BPA”), its synonyms and/Chemical Abstracts Service (CAS) registry number, with and without chemical class name (eg, “BPA + bisphenol”), combined with terms including but not limited to “use*,” “application*,” “*production*,” “manufactur*,” etc. We aggressively sought industry sources as they frequently contain the most detailed and quantitative assessments of chemical applications.

To qualify as a plastic-related use of a chemical, an application must belong in whole or in part to at least one of the following categories (based on previously published work [12]):

1. Monomers: used to make synthetic or semisynthetic polymers
2. Plastic additives: added to polymers to enhance desirable characteristics. Here we include functional additives (stabilizers, antistatic agents, flame retardants, plasticizers, lubricants, slip agents, curing agents, foaming agents, biocides); colorants (pigments, azodyes); fillers (carbon black, talc, calcium carbonate); and reinforcements (glass and carbon fibers)
3. Processing aids: enable or ease the production or processing of plastics (polymerization catalysts, solvents, mold release agents, lubricants)
4. Surface treatments applied to plastics as defined earlier (paints, adhesives, coatings; many of these are synthetic polymeric materials themselves)

Due to a lack of available data for perfluorooctanoic acid (PFOA), we used percentage of emissions by source as a proxy for percentage use for different applications.

For each source containing information identifying a chemical's uses by proportion, we classified whether the use the following:

1. fully meets the criteria for plastic-related use;
2. partially meets the criteria for plastic-related use; (ie, there is evidence that the use sometimes meets the criteria listed earlier as plastic related, but there are some applications for which it does not meet the criteria; or there was insufficient evidence to say unequivocally that it always meets the criteria)

or

(3) fully falls outside the criteria for a plastic-related use. (ie, there is either evidence that explicitly states that this use never meets the criteria listed earlier as plastic related; or no evidence is found of the use qualifying as plastic-related applying the aforementioned criteria)

For categories that partially meet the criteria, we first attempted to determine what part of the fraction was plastic related based on secondary sources. If that determination was not possible, we assumed that half of the application was plastic related, using 25% (low-end estimate) or 75% (high-end estimate) in sensitivity analyses.

Some chemicals had more than one source that could be used to derive a PRF. To determine which PRF we used for subsequent analyses, we prioritized sources that relied on use data collected between 2000 and 2010, as these years are most relevant to the 2018 disease burden estimates. Among these, if multiple sources were found, we prioritized US-based plastics and chemical industry sources over all others (as they are most relevant to our study and likely represent the most comprehensive sources), followed by (in declining order of priority): US government sources, international industry sources, international government sources, and other authoritative publications.

### Aggregating Plastic-attributable Disease Burden and Cost Estimates

To calculate base case estimates, we multiplied base case PRFs by base case estimates of disease burden and cost estimates from the primary manuscripts. For example, our previous study [6] estimates the total estimated cost of bisphenol A (BPA) exposure as $1.04 billion. We determine that 98% of all BPA is for plastic-related use. Therefore, we assume that 98% of BPA exposure can be attributed to its uses in plastics. We also assume that disease burden is directly proportional to exposure. Therefore, the plastic-related health burden of BPA is $1.02 billion (0.98 × 1.04 billion). Our estimates of PFAS-related disease were based on PFOA, so we applied PFOA-related PRFs in main estimates to disease burden and cost estimates based on PFOA exposure for main estimates of PFAS-related plastic disease burden and costs. Our previous estimates of phthalate-induced infertility were based on effects of benzyl butyl phthalate (BBP) and dibutyl phthalate (DBP) exposures. To calculate the plastic-related proportion of disease burden and costs, we used DBP PRFs as they comprised the majority of the infertility burden identified.

We also performed a multiway sensitivity analysis to provide a range that represents uncertainties from the multiple sources of data. Low-end estimates used the lowest end of PRF ranges by the lowest end of disease burden and cost estimates, similarly high-end estimates were generated by applying the highest available PRFs by the highest end of the disease burden and cost estimate ranges. PFOS-derived PRFs were considered for outcomes attributable to PFAS, and benzyl butyl phthalate-PRFs were used for infertility attributable to phthalates.

## Results

### Estimates of Plastic-related Fractions by Chemical

For BPA (Table 1), we identified 97.5% of exposure and therefore disease burden and costs to be due to plastic, with a range of 96.25% to 98.75% for sensitivity analysis. Polycarbonate plastic was estimated to comprise 65% of BPA use, with another 30% of BPA use for epoxy resins [13]. For these uses, the entirety of BPA exposure is plastic related. For the remaining 5%, BPA was identified as being used for other applications including as a stabilizer and an antioxidant in the production of PVC, and as a precursor in the manufacturing of a brominated flame retardant, tetrabromobisphenol A (TBBPA) [13]. Because TBBPA is primarily used as a flame-retardant additive not only in plastics, but also paper and textiles [14], and no data sources were identified to quantify more accurately the percentage of BPA use for plastic in this category, base case analyses assumed 50% use for plastic with sensitivity analyses adopting a 25% to 75% range.

**Table 1.** Plastic-related use of bisphenol A

Use	% Total use	PRF main estimate	PRF low estimate	PRF high estimate	Comments
Polycarbonate plastic	65%	100%	100%	100%	All plastic related
Epoxy resins	30%	100%	100%	100%	All plastic related
Other	5%	50%	25%	75%	Partially plastic related, including BPA as stabilizer and an antioxidant in the production of PVC and as a precursor in the manufacturing of a brominated flame retardant, TBBPA. TBBPA is primarily used as a flame retardant in additive to plastics, but also paper and textiles
All uses	100%	98%	96%	99%	

Abbreviations: BPA, bisphenol A; PRF, plastic-related fraction; PVC, polyvinyl chloride; TBBPA, tetrabromobisphenol A.

While other sources provide lists of uses of BPA [15], the study by Vasiljevic and Harner [13] was the only source we could identify that explicitly provided a breakdown by percentage of different uses.

For bis(2-ethylhexyl)phthalate (DEHP) (Table 2), we identified 98.5% of exposure and therefore disease burden and costs to be due to plastic, with a range of 98% to 99% for sensitivity analysis. Use of DEHP as a plasticizer for polymers, primarily PVC plastic, was estimated to comprise 97% of its use [16]. For this use, the entirety of DEHP exposure is plastic-related. For the remaining 3%, DEHP is used in other applications including adhesives and sealants, lacquers and paints, printing inks for paper and plastics, printing inks for textiles, rubber and ceramics for electronic purposes, and dielectric fluid in capacitors [16]. We could find no evidence to suggest that ceramics and dielectric fluids are ever plastics related. For others, including adhesives and sealants [17], lacquers and paints, printing inks [18], and applications in rubber [19], we found evidence that they sometimes meet our criteria for plastics-related use; but we could not conclusively determine whether all applications using DEHP were plastics related for these categories. Because no data sources were identified to quantify more accurately the percentage of DEHP for plastic-related use in these categories, in base case analyses we assumed 50% use for plastic, with sensitivity analyses adopting a 25% to 75% range. The ECHA report provided the only breakdown by percentage of different uses that we could identify.

**Table 2.** Plastic-related use of di-2-ethylhexylphthalate

Use	% Total use	PRF main estimate	PRF low estimate	PRF high estimate	Details
Plasticizer in polymer (primarily PVC)	97%	100%	100%	100%	All plastic related
Other	3%	50%	25%	75%	Partially plastic related Includes adhesives and sealants, lacquers and paints, printing inks for paper and plastics, printing inks for textiles, rubber and ceramics for electronic purposes, dielectric fluid in capacitors
All uses	100%	98.5%	98%	99%	

Abbreviations: PRF, plastic-related fraction; PVC, polyvinyl chloride.

For BBP (Table 3), we identified 100% of exposure and therefore disease burden and costs to be due to plastic, with a range of 100% to 100% for sensitivity analysis. PVC and polyvinyl acetate (PVAc) applications were estimated to comprise 100% of BBP use. Specifically, 40% of BBP was used for PVC flooring, in the form of sheet and tile. The remaining 55% was used in PVAc, a polymeric adhesive used in wood glue and other adhesives, caulks, and sealants [20]. Both of these fully meet our definition for plastic related (as additives). As no other uses for BBP were recorded and all of the reported uses were plastic related, base case analyses assumed 100% of all BBP use as plastic related.

**Table 3.** Plastic-related use of benzylbutylphthalate

Use	% Total use	PRF main estimate	PRF low estimate	PRF high estimate	Details
PVC flooring (sheet and tile)	40	100%	100%	100%	All plastic related
PVAc-based caulks and sealants	35	100%	100%	100%	All plastic related
PVAc-based adhesives	25	100%	100%	100%	All plastic related
Total PRF	100%	100%	100%	100%	All plastic related

Abbreviations: PRF, plastic-related fraction; PVC, polyvinyl chloride; PVAc, polyvinyl acetate.

We identified one other source providing a breakdown by percentage of different uses, an ECHA report on BBP [21] reporting that 70% of BBP is used as a polymer plasticizer, primarily for PVC flooring applications. It also notes that industry sources indicate more than 90% of BBP is used for plasticizing PVC and other polymers. The original web-based source for this industry value is no longer available. We use the Bizzari values here as it is an established industry source and therefore most relevant our study [20].

For DBP (Table 4), we identified 85% of exposure and therefore disease burden and costs to be due to plastic, with a range of 79% to 91% for sensitivity analysis, using total tons per year by source reported in ECHA's 2009 report on DBP [22]. Polymer formulation and processing was estimated to comprise 71% of DBP use. Another 2% was reported as used in nitrocellulose lacquer-based paints, which are polymeric. For these two uses the entirety of DBP exposure is plastic related. Additionally, DBP is used in adhesives (23%) and grouting agents (1%). While the types of adhesives and grouting agents that DBP is used for are typically polymeric (PVA based and PUR based, respectively), we could not conclusively quantify the percentage of DBP for plastic-related uses in these categories, so base case analyses assumed 50% use for plastic with sensitivity analyses adopting a 25% to 75% range. A total of 3% of all DBP use is estimated to be nonpolymeric, and is associated with its use as a solvent for oil-soluble organic compounds including some dyes and insecticides.

**Table 4.** Plastic-related use of dibutylphthalate

Use	Tons/y in source	% Total use	PRF main estimate	PRF low estimate	PRF high estimate	Details
Polymer formulation and processing	5900	71.26%	100%	100%	100%	All plastic related Includes flooring, garden hoses, automotive applications, fiberglass, and others
Paints (nitrocellulose lacquers)	160	1.93%	100%	100%	100%	All plastics related Nitrocellulose lacquers are a polymeric substance
Adhesives	1890	22.83%	50%	25%	75%	Partially plastics related Includes adhesives in paper and packaging, construction, automotive applications
Grouting agents	80	0.97%	50%	25%	75%	Partially plastics related Used in construction work
Other, nonpolymeric	250	3.02%	0%	0%	0%	Not plastics related Includes solvent for oil-soluble dyes, insecticides, peroxides, and other organic compounds
Total PRF	8280	100%	85%	79%	91%	

Abbreviation: PRF, plastic-related fraction.

The 2004 European Chemicals Bureau Report [23] presents similar manufacturing-related data as a later ECHA report from 2009 [22], both relying on European Union (EU) industry-reported manufacturing data from 1997. No additional studies could be obtained.

For PFOA (Table 5) we identified 93% of exposure and therefore disease burden and costs to be due to plastic, with a range of 89.55% to 96.45% for sensitivity analysis. Data to determine percentages of PFOA used for different applications were not available, so we use emissions associated with ammonium perfluorooctanoate (APFO) reported by Prevedouros et al [24]. PFOA is used and produced in the form of APFO; APFO is also sometimes used synonymously with PFOA [25].

**Table 5.** Plastic-related use of perfluorooctanoic acid

Emissions source	% of total PFCA-related emissions	% out of total emissions attributed to APFO/PFOA	PRF main estimate	PRF low estimate	PRF high estimate	Details
APFO used in fluoropolymer manufacture	57%	79.2%	100%	100%	100%	All plastics related
APFO manufacture	10%	13.8%	50%	25%	75%	Partially plastics related (includes nonplastic-related emissions from applications such as such as AFFF)
APFO for fluoropolymer dispersions	5%	6.9%	100%	100%	100%	All plastic related
Total PRF	72%	100%	93%	90%	96%	

Abbreviations: AFFF, aqueous film forming foams; APFO, ammonium perfluorooctanoate; PFCA, perfluorocarboxylic acids; PFOA, perfluorooctanoic acid; PRF, plastic-related fraction.

Emissions from APFO used in fluoropolymer manufacture was estimated to comprise 79.2% of all emissions associated with APFO production and use. A further 6.9% of emissions are attributed to APFO used in fluoropolymer dispersions, which are a polymeric use. For these two uses, the entirety of APFO/PFOA exposure is plastic related. Additionally, the manufacture of APFO is responsible for 13.8% of total APFO-related emissions. We could not conclusively quantify the percentage of APFO for plastic-related uses in this category, so base case analyses assumed 50% use for plastic with sensitivity analyses adopting a 25% to 75% range.

The work by Prevedouros et al in 2006 [24] yielded the only data that allowed us to estimate APFO/PFOA use or emissions by application.

For PFOS (Table 6), we identified 48% of exposure and therefore disease burden and costs to be due to plastic, with a range of 24% to 72% for sensitivity analysis, using total metric tons by source reported in Lim et al [26]. These data are republished from a 2000 technical report by the 3M company [22].

**Table 6.** Plastic-related use of perfluorooctane sulfate acid

Use	Metric tons in source	% Total use	PRF main estimate	PRF low estimate	PRF high estimate	Details
Surface treatments	2160	48.20%	50%	25%	75%	Partially plastic related. Includes textile mills, leather tanneries, finishers, fiber producers, carpet manufacturers, apparel and leather, upholstery, carpet, and automobile interiors
Paper protection	1490	33.25%	0%	0%	0%	Not plastic related. Food contact applications (plates, food containers, bags, and wraps), nonfood contact applications (folding cartons, containers, carbonless forms, masking papers)
Firefighting foams	151	3.37%	0%	0%	0%	Not plastic-related.
Other performance chemicals	680	15.18%	50%	25%	75%	Partially plastic related Mining and oil well surfactants, acid mist suppressants for metal plating, electronic etching baths, photolithography, electronic chemicals, hydraulic fluid additives, alkaline cleaners, floor polishes, photographic film, denture cleaners, shampoos, chemical intermediates, coating additives, carpet spot cleaners, insecticide in bait stations
Total PRF	4481	100%	31.7%	15.8%	47.5%	

Abbreviation: PRF, plastic-related fraction.

Three use categories listed in the report are partially plastic related. A total of 48.20% of PFOS use was identified as being used for surface treatments, some of which are likely to be plastic related. Specific uses of surface treatments include use in carpet, apparel, and upholstery, all of which are textile based. While exact numbers were not available, industry reporting on fiber production suggests that synthetic textiles account for 64% [27] to 91% [28] of all textiles produced globally. Therefore, we assume that some PFOA surface treatments are used for synthetic textiles that are plastic-related applications. Another 15% of PFOS was identified as being used for “other performance chemicals,” including floor polishes (which are primarily polymeric, to which PFOS is added for improving performance and enhancing gloss [29]). PFOS “coating additives” may also be used in polymeric coatings [30]. Others among the specific uses listed under “other performance chemicals” are nonpolymeric (eg, some surfactants, cleaning agents, and insecticides).

As we could not quantify more accurately the percentage of PFOS use for plastic in the categories of surface treatments, paper protection, and other performance chemicals, base case analyses assumed 50% use for plastic for each of these categories, with sensitivity analyses adopting a 25% to 75% range.

An additional 33% of PFOS is used for paper production. While paper products often contain plastic or polymeric components, PFOS (or other PFAS) appear to be found primarily or exclusively in paper products that do not include a plastic layer [31]. Therefore, we assume that PFOS for paper production does not include plastic-related uses. Fire-fighting foams, which comprise 3% of PFOS use, do not meet our criteria for plastic-related uses.

For PBDE-47, we identified 98.25% of exposure and therefore disease burden and costs to be due to plastic, with a range of 97.38% to 99.13% for sensitivity analysis. PBDE-47 as a commercial product was exclusively found in penta-BDE and was used as a proxy for PBDE-47 use [32].

The manufacture of flexible PUR foams (FPUF), a polymeric material, was reported to comprise 95% to 98% of penta-PBDE use [32]. Of the remaining 2% to 5%, an unspecified “small percentage” was used for commercial adhesive products. At least one source suggests that these adhesives are polymeric [33]. We therefore assume that this category is at least partially plastic related. Penta-PBDE may have had additional historical uses. Use in textile coatings, PUR electronics coatings, and “fluids used in hydraulic and oilfield completion” are reported [34], but these uses appear to have been discontinued by 1999 or 2001 [32, 35] and we could not find reporting to quantify how much Penta-PBDE was applied to these uses. It is worth noting that these quantities were likely very small in comparison with penta-PBDE's use in FPUF; the EU risk assessment report for penta-BDE [35] assumes that all penta-PBDE used in the EU was used for PUR foam and that other uses were negligible, based on use data from industry.

Carrying forward the PRFs, and updating previous estimates of disease burden and costs (Table 7), we estimate $249 billion (sensitivity analysis: $226-$289 billion) in plastic-attributable disease burden in 2018. The largest proportion of these costs arose from PBDE exposure ($159 billion, sensitivity analysis: $157 billion-$161 billion), though $66.7 billion ($64.7 billion-67.3 billion) was due to phthalate exposure and $22.4 billion was due to PFAS exposure (sensitivity analysis: $3.85 billion-$60.1 billion) (Table 8).

**Table 7.** Plastic-related use of 2,2′4,4′-tetrabromodiphenyl ether

Use	% Total use	PRF main estimate	PRF low estimate	PRF high estimate	Comments
Manufacture of FPUF	96.5% (95%-98%)	100%	100%	100%	All plastic related. Primarily used in furniture industry
Commercial adhesive products, other uses	3.5%	50%	25%	75%	Partially plastic related
All uses	100%	98.25	97.38	99.13	

Abbreviation: FPUF, flexible polyurethane foams; PRF, plastic-related fraction.

**Table 8.** Estimates of plastic-attributable disease burden and costs

Exposure	Life stage of exposure	Outcome	Cost of illness across exposure routes (source [6] unless otherwise indicated)	PRF applied in base case (sensitivity analysis)	Plastic-related cases (sensitivity analysis)	Plastic-related cost of illness (sensitivity analysis)
PBDE-47	Prenatal	IQ points loss and intellectual disability	$162 billion	98% (97%-99%)	713 000 IQ points lost 23 900 cases of intellectual disability (23 700-24 100)	$158 billion ($157-160 billion)
	Prenatal	Cryptorchidism	$35.7 million		4200 cases (4200-4300)	$35.0 million ($34.6-35.3 million)
	Prenatal	Testicular cancer	$81 million		3500 cases (3500-3600)	$79.4 million ($78.6-80.2 million)
	All	All	$162 billion		N/A	**$159 billion ($157-161 billion)**
DEHP	Women	Obesity	$1.95 billion	98% (96%-99%)	5350 cases (5250-5410)	$1.92 billion ($1.88-1.94 billion)
		Type 2 diabetes	$259 million		2870 cases (2810-2900)	$253 million ($249-256 million)
	Women	Endometriosis	$39.7 billion		59 100 cases (57 900-59 700)	$38.9 billion ($38.1-39.3 billion)
	Adults	Cardiovascular Mortality	$23.8 billion [7]		50 200 cases (49 200-50 700)	$23.4 billion ($22.9-23.6 billion)
BBP and DBP	Men	Male infertility	$3.14 billion	100% (71%-100%)	121 000 cases (85 900-121 000)	$3.14 billion ($1.59-2.24 billion)
Total phthalates	All	All	$68.9 billion	N/A	N/A	**$66.7 billion ($64.7-67.3 billion)**
BPA	Prenatal	Childhood obesity	$1.04 billion	98% (96%-99%)	7130 (6850-7060)	**$1.02 billion ($1.00-1.03 billion)**
PFAS (PFOA in main estimates, PFOS in sensitivity analysis)	Prenatal	Low birth weight	$1.42 billion [8]	93% (16%-96%)	9350 cases (1610-93 000)	$1.32 billion ($227 million-13.2 billion)
	Prenatal	Childhood obesity	$2.65 billion [8]		118 000 cases (20 400-444 000)	$2.46 billion ($424 million-$9.22 billion)
	Children	Pneumonia	$1.49 million [8]		415 cases (72-6490)	$1.39 million ($238 000-$21.6 million)
	Pregnant people	Gestational diabetes	$414 million [8]		5640 cases (970-12 000)	$385 million ($66.2-$818 million)
	Adult	Obesity	$17.0 billon [8]		3 990 000 cases (687 000-4 120 000)	$15.8 billion ($2.72-$16.3 billion)
	Adult	Kidney cancer	$180 million [8]		132 cases (23-136)	$171 million ($29.4-$177 million)
	Adults	Couple infertility	$37.6 million [8]		551 cases (95-25 100)	$35.0 million ($6.06 million-$1.59 billion)
	Women	Hypothyroidism	$1.26 billion [8]		13 600 cases (2300-57 400)	$1.17 billion ($201 million-4.98 billion)
	Women	Type 2 diabetes	$140 million [8]		1600 cases (276-1660)	$130 million ($22.4-$134 million)
	Women	Endometriosis	$397 million [8]		647 cases (111-17 300)	$369 million ($63.5 million-$9.79 billion)
	Women	Polycystic ovary syndrome	$10.5 million [8]		6700 cases (1150-7200)	$9.77 million ($1.68-$10.5 million)
	Women	Breast cancer	$555 million [8]		392 cases (101-2971)	$516 million ($133 million-$3.92 billion)
	Men	Testicular cancer	$6.85 million [8]		5 cases (1-5)	$6.37 million ($1.10-$6.58 million)
	All	All	$24.1 billion [8]		N/A	**$22.4 billion ($3.85-60.1 billion)**
All	All	All		N/A	N/A	**$249 billion ($226-**$**289 billion)**

Abbreviation: BBP, butyl benzyl phthalate; BPA, bisphenol A; DBP, dibutyl phthalate; DEHP, bis(2-ethylhexyl)phthalate; N/A, not available; PBDE, polybrominated diphenyl ether; PFAS, perfluoroalkyl and polyfluoroalkyl substances; PFOA, perfluorooctanoic acid; PFOS, perfluorooctane sulfonate; PRF, plastic-related fraction.

## Discussion

In the present study, we quantify high PRFs for some but not all of the most prevalent exposures for chemicals used in plastic materials. The disease burden directly attributable to plastic production and consumption is substantial, and runs across the entire lifespan. We also identify billions of dollars in annual costs directly attributable to plastic uses, driven largely by PBDEs. These costs should be considered alongside the costs of safer alternatives.

As a first effort to quantify the proportion of disease and disability directly due to plastics in the United States, our analysis is limited by many factors. We appreciate the limits of the materials reviewed to derive PRFs. Detailed descriptions of chemicals’ applications from North American sources obtained during the years that are most relevant to exposure would have allowed for more accurate estimates. We would prefer to have comprehensive data on exposure patterns and sources to better inform the analyses. Further work can hone these estimates, and specify food packaging and other origins of plastic-related diseases and dysfunctions.

We were able to estimate disease burden for only a few chemicals used in plastic materials, and a subset of diseases for those few chemicals. A recent review identified probable contribution of phthalates to preterm birth, reduced anogenital distance in boys, reduced sperm count and function, and childhood obesity; PFAS to impaired glucose tolerance in pregnancy; and BPA and bisphenol S to adult diabetes [36]. We also note that the majority of previous studies focused on endocrine-related diseases and dysfunctions, when PFAS and other chemicals used in plastic materials can impair renal function [37-39]. Table 8 [11] presents a list of other chemicals used in plastics (adapted from [12]) that are included in the National Health and Nutrition Examination Survey's biomonitoring program. Many more chemicals of concern have been identified [2], with an even broader array of potential consequences that may emerge through the march of scientific investigation and understanding.

We can draw only limited comparisons to the calculations of the Minderoo-Monaco Commission due to the lack of a detailed methods section in its report [9], despite its use of our work [6-8]. Our work corrects an important mathematical error in the commission's report that could mislead and overestimate mortality due to phthalates [7, 40]. The commission also failed to quantify disease burden and costs due to PFAS exposures from plastic materials, which contribute $22.4 billion to our estimate. The commission applied a $5.4 million value of a statistical life (a willingness-to-pay estimate [41]) for each case of phthalate-induced cardiovascular mortality, rather than the $439 313 lost economic productivity (indirect costs of mortality using a cost-of-illness approach) that we applied [7]. We do not dispute the value of willingness-to-pay approaches; debates about which approach to apply abound in the economic and public health literature [42]. Willingness-to-pay and cost-of-illness approaches in theory should converge, with the latter method underestimating in real-life practice due to limited available estimates of costs to society [43]. Notably, had we applied value of a statistical life due to phthalate-induced mortality as the commission did, the health costs due to plastic would have been $269 billion higher, with total costs exceeding $500 billion. The chief driver of health costs would then have been phthalates rather than PBDEs.

Having addressed important limitations, the present work has many strengths including the use of a rigorous methodology for estimating the environmental burden of disease first described by the Institute of Medicine [44], and which remains highly informative in public health and policy-making. The methodology used here can be extrapolated to global burdens of disease [45], which still do not incorporate sufficiently environmental exposures with substantial evidence for causation [46].

We conclude that the Global Plastics Treaty should reduce the use of chemicals of concern, particularly PFAS, bisphenols, flame retardants, and phthalates. The benefits to these reductions are substantial, as reduced exposures will lead to savings in health-care costs due to lower disease burdens. These benefits in the United States alone are likely to be in the billions of dollars and accrue annually as sustained reductions in exposures are achieved. PBDEs are the largest driver of social costs in the United States, followed by phthalates. Actions to address these chemicals through the Global Plastics Treaty and country-specific regulatory interventions will produce benefits directly in relationship to reductions in specific exposures produced.

## Data Availability

Original data generated and analyzed during this study are included in this published article or in the data repositories listed in “References.”

## Abbreviations

AFFF: aqueous film forming foam
APFO: ammonium perfluorooctanoate
BBP: butyl benzyl phthalate
BPA: bisphenol A
DBP: dibutyl phthalate
DEHP: bis(2-ethylhexyl)phthalate
ECHA: European Chemicals Agency
EU: European Union
FPUF: flexible polyurethane foams
N/A: not available
PBDE: polybrominated diphenyl ether
PFAS: perfluoroalkyl and polyfluoroalkyl substances
PFCA: perfluorocarboxylic acids
PFOA: perfluorooctanoic acid
PFOS: perfluorooctane sulfonate
PRF: plastic-related fraction
PUR: polyurethane
PVAc: polyvinyl acetate
PVC: polyvinyl chloride
TBBPA: tetrabromobisphenol A
