# Correction to: “Chemicals Used in Plastic Materials: An Estimate of the Attributable Disease Burden and Costs in the United States”

**DOI:** 10.1210/jendso/bvae019

**Published:** 2024-02-14

In the above-named article by Trasande L, Krithivasan R, Park K, Obsekov V, and Belliveau M (*J Endo Society*. 2024; 8(2); doi: 10.1210/jendso/bvad163), there was an error in Reference 11:

In the originally published version of this manuscript, in Reference 11, details of the repository for the supplemental material were omitted: “Trasande L, Krithivasan R, Park K, Obsekov V, Belliveau M. Chemicals used in plastic materials: an estimate of the attributable disease burden and costs in the US: supplemental material 2023.” In the corrected article, Reference 11 reads: Trasande L, Krithivasan R, Park K, Obsekov V, Belliveau M. Chemicals used in plastic materials: an estimate of the attributable disease burden and costs in the US: supplemental material 2023. Accessed 31 January 2024. https://www.researchgate.net/publication/377491167_Supplementary_Material_for_Chemicals_Used_in_Plastic_Materials_An_Estimate_of_the_Attributable_Disease_Burden_and_Costs_in_the_US?channel=doi&linkId=65a9a8ebf323f74ff1cc2d1a&showFulltext=true#fullTextFileContent.”

The article has been corrected online.

doi: 10.1210/jendso/bvad163

